# News and Coming Events

**Published:** 1908-09-12

**Authors:** 


### News and Coming Events.

Dr. Francis James Henderson Coutts, M.D., B.Sc., D.P.H.Manch., has been appointed an Inspector of Foods on the medical staff of the Local Government Board.

Professor Robert Koch has been appointed by the German Government to attend the International Tuberculosis Congress at Washington as its representative.

Among the latest contributions to King Edward's Hospital Fund for London is the sum of £105, being the annual subscription of his Majesty the King, founder and patron of the Fund.

Lieut.-Col. John Lawrence Macpherson, R.E. (retired), of Hastings, left £500 each to Guy's Hospital, London Hospital, St. Thomas's Hospital, the British Home and Hospital for Incurables, and the Central London Throat and Ear Hospital.

The medical session at the University of Manchester will be inaugurated by an address delivered on October 1, at 4.30 p.m., by Sir Thomas Clifford Allbutt, K.C.B., M.A., M.D., D.Sc., LL.D., F.R.S., Regius professor of physic, University of Cambridge.

Dr. A. M. Fleming, C.M.G., Dr. Walter M. Eaton, and Dr. F. Heygate Ellis have been appointed medical inspectors in the service of the British South Africa Company. Dr. Fleming has occupied the offices of medical director to the company and principal medical officer of the British South Africa Police.

The second Congress of the International Society of Surgery will be held in Brussels from September 21 to 25, under the presidency of Dr. Von Czerny, of Heidelberg. The subjects selected for discussion are : The Nature and treatment of Cancer, the Diseases of the Liver and Bile Ducts, the Methods of Inducing Anæsthesia, the Ætiology and Treatment of Hernia, and the Surgery of the Spinal Cord. In connection with the museum, a special effort has been made to collect everything relating to cancer, and there will be the usual exhibition of surgical instruments and appliances.

On September 4 a special meeting of the Senate of Dublin University was held in the Regent House, Trinity College, for the purpose of conferring honorary degrees on certain members of the British Association. The degree of D.Sc. was conferred upon Dr. Charles Scott Sherrington, F.R.S., Professor of Phy[illegible]ology in the University of Liverpool, and Dr. Archi[illegible]ald Byron Macallum, Professor of Physiology in the University of Toronto; while the degree of M.D. was con[illegible]erred upon Sir Thomas Lauder Brunton, Bart., F.R.S.

Mr. John Capel Hanbury has given £2,000 to the Pontypool Hospital for the erection of a new wing in memory of his son.

The death occurred on August 30, at the age of forty-six, of Dr. Montague Williams Oldham, M.R.C.S., L.R.C.P. (Lond.), M.D. (Brux.), M.S.A., for eleven years hon. surgeon Mansfield Hospital, late surgeon Coventry Provident Dispensary, and house surgeon Burton-on-Trent Infirmary.

A special meeting of the International Council of Women was opened on August 31, in Geneva, by Lady Aberdeen, who is acting president of the Congress. The council consists of delegates from the different national councils, which, in their turn, are federations of women's societies in the United States, Canada, Germany, Sweden, Great Britain, and Ireland, Denmark, the Netherlands, New South Wales, Tasmania, Italy, France, Switzerland, South Australia, Austria, Norway, Belgium, Queensland, Bulgaria, and Greece. The ladies are all engaged in some distinct line of work, such as the education of women, sanitary matters, and hygiene.

At a meeting of the Board of the London Temperance Hospital on September 1 it was unanimously agreed, on the motion of the Chairman, "That in deeply sympathising with the bereaved family of the late Canon Fleming, B.D., we deplore the great public loss entailed by the removal of one who was endeared to us by numerous acts of kindness to this institution during the last thirty-five years. By his death the hospitals of London are deprived of an eloquent advocate and powerful supporter. As a temperance reformer and Christian philanthropist he leaves a distinguished record of fidelity and zeal, and will be long remembered as a bright example of that pure and undefiled religion which consists in love and service to man as evidence of love and devotion to God."

On September 3rd 350 German physicians landed at Ryde Pier from the steamer *Oceana,* on their annual tour. After inspecting the Royal County Hospital the party proceeded by special train to Ventnor, where the streets were decorated in their honour, and they were received by the Municipal authorities. They visited the various hygienic establishments, and at the Royal National Home for Consumption were welcomed by the medical staff and local committee. An address descriptive of the cure pursued at this national institution was given by Dr. Robertson, the senior visiting physician. Luncheon was served at the Grand Pavilion, where Professor Rosin afterwards gave an address on sea sickness. A lecture was delivered in the Town Hall by Lieut. Damant, R.N., on caisson disease. The visitors proceeded by special train to Osborne.

At Birmingham, on September 2, Harley Lord, who described himself as a tooth specialist, was summoned at the instance of the British Dental Association for holding himself out to be a qualified practitioner registered under the Dentists Act. Mr. Sandlands, who prosecuted, said the Dental Association was of opinion that the public ought to be enabled to distinguish between men who were properly qualified to practise dentistry and those who practised without being so qualified. An example ought to be made in order to call attention to the matter. The defendant was not registered, but the description in the advertisement to which the summons referred implied that he was specially qualified to practise dentistry, and that constituted an offence under the Dentists Act. A fine of £5 and five guineas costs was imposed.

Last year, out of a total delivery of 417,057 tons of meat at the London Central Markets 1,038 tons were condemned as unfit for human food. Of that quantity 174 tons were imported frozen and chilled meat. Upwards of 89 tons of the seizures were on account of tuberculosis, these including 2,309 pigs. The total seizures represented one ton in every 401 tons delivered. At Billingsgate Market, out of a total delivery of 233,715 tons of fish, 783 tons were seized—or one ton in every 297 tons delivered. At the Smithfield Fish Market, out of 1,876 tons of fish received, four tons were condemned. At riverside wharves 194 tons of unsound food were removed and destroyed—41 tons being tinned fruit and vegetables, 67 tons tinned milk, 34 tons miscellaneous tinned food, 16 tons fruit pulp, and 30 tons fruit and vegetables. The greater portion of this was removed at the written request of the owners under the Public Health (London) Act, 1891, as trade refuse.

Sir Alfred Jones (chairman of the Liverpool School of Tropical Medicine) is making arrangements to despatch an expedition to Jamaica to investigate tropical diseases there and the insect life of the island, which is responsible for carrying disease. It is intended to send Mr. Robert Newstead, the lecturer in Economic Entomology and Parasitology of the Liverpool School of Tropical Medicine to Jamaica in the first week of November to undertake the investigation of the ticks there responsible for certain diseases in animals, and of disease-bearing insects. He may be accompanied by a medical research investigator, whose duties would be to investigate indigenous diseases of the island. Mr. Newstead was director of the Grosvenor Museum, Chester, for nearly twenty years, and in April 1905 was appointed lecturer in Economic Entomology and Parasitology at the University of Liverpool in the Liverpool School of Tropical Medicine.

The President of the Local Government Board has arranged for the making of the following additional researches in connection with the annual grant voted by Parliament in aid of scientific investigations concerning the causes and processes of disease: (1) A chemical and bacteriological investigation by Mr. C. G. Moor, M.A., F.I.C., and Dr. Hewlett, Professor of Pathology at King's College, London, as to the influence of softening and of other chemical processes on the purity of water supplies from the chalk as shown in actual experience and under experimental conditions; (2) An investigation by Professor Sidney Martin, F.R.S., into the powers of production of disease possessed by certain streptococci and by the poisonous substances produced by them, in continuance of previous investigations by him on the same subject. These two investigations complete the allocation of the Scientific Grant for the year 1908-9.

The Hostel of St. Luke (the Clergy Nursing Home, 14 Fitzroy Square, London, W.) has been reopened for the reception of patients after completion of some building work and cleaning. Mr. H. S. Collier, F.R.C.S., Mr. H. Fairbank, M.S., F.R.C.S., Dr. N. I. C. Tirard, and Mr. Ernest Waggett have been appointed on the staff of physicians and surgeons to the hostel. The hon. treasurer, Mr. A. H. Tritton, makes an earnest appeal for donations to the building fund to pay off the amount still due to the bankers, also for increased subscriptions to the general fund for maintenance.

The annual conference of the Sanitary Inspectors' Association opened on Sept. 8 at Liverpool. There were 450 delegates present. After the reception of members and delegates by the Lord Mayor (Dr. Caton), Sir James Crichton-Browne delivered his presidential address. A paper was read by the Lord Mayor on "Sanitation in Ancient Greece," and the delegates were entertained to luncheon by the Lord Mayor and to tea by the City Council. On Sept. 9 papers were read by Sir Rubert Boyce (Liverpool University) on "Organised Endeavour against Diseases, or Lessons to be Learnt from the Health Campaign in Ireland"; and by Dr. A. A. Mussen on "Housing and Town Planning." Papers were read on Sept. 10 by Dr. W. Hanna (Liverpool) on "Supervision of our Imported Food Supply"; and by Mr. A. E. Hudson on "Administration Difficulties in Connection with Meat and Milk."

